# Antroquinonol Exerts Immunosuppressive Effect on CD8^+^ T Cell Proliferation and Activation to Resist Depigmentation Induced by H_2_O_2_

**DOI:** 10.1155/2017/9303054

**Published:** 2017-12-31

**Authors:** Cuiping Guan, Qingtian Li, Xiuzu Song, Wen Xu, Liuyu Li, Aie Xu

**Affiliations:** ^1^Department of Dermatology, The Third People's Hospital of Hangzhou, Hangzhou 310009, China; ^2^Department of Medicine, Baylor College of Medicine, Houston, TX 77030, USA

## Abstract

Antroquinonol was investigated as antioxidant and inhibition of inflammatory responses. Our study was to evaluate its immunosuppressive effect on CD8^+^ T cells and protective effect on depigmentation. CD8^+^ T cells were treated with antroquinonol *in vitro*, and C57BL/6 mice were treated with antroquinonol with or without H_2_O_2_ *in vivo* for 50 consecutive days. We found antroquinonol could inhibit proliferation of CD8^+^ T cells and suppress the production of cytokines IL-2 and IFN-*γ* and T cell activation markers CD69 and CD137 *in vitro*. H_2_O_2_ treatment induced depigmentation and reduced hair follicle length, skin thickness, and tyrosinase expression *in vivo*. Whereas, antroquinonol obviously ameliorated depigmentation of mice skin and resisted the reduction of hair follicle length, skin thickness, and tyrosinase expression induced by H_2_O_2_. Antroquinonol decreased CD8^+^ T cell infiltration in mice skin, inhibited the production of IL-2 and IFN-*γ*, and decreased the expression of CXCL10 and CXCR3. Summarily, our data shows antroquinonol inhibits CD8^+^ T cell proliferation *in vitro*. It also reduces CD8^+^ T cell infiltration and proinflammatory cytokine secretion and suppresses the thinning of epidermal layer *in vivo*. Our findings suggest that antroquinonol exerts immunosuppressive effects on CD8^+^ T cell proliferation and activation to resist depigmentation induced by H_2_O_2_.

## 1. Introduction

Vitiligo is a common dermatological disorder characterized by the progressive depigmentation caused by a loss of melanocytes in the epidermis. Absence of melanocytes in the skin lesion has been considered as a core event in the pathogenesis of vitiligo [1]. A single dominant pathway appears unable to explain all causes of vitiligo. Obviously, loss of melanocytes in vitiligo seems to occur through a complex interaction of several mechanisms including environmental, biochemical, immunological, and genetic events that act in concert [2]. In vitiligo epidermis, the increased levels of reactive oxygen species (ROS) were observed [3, 4]. −89 A/T polymorphisms of catalase in vitiligo patients showed significantly increased lipid peroxidation levels [5]. Increased malondialdehyde and decreased catalase were found in vitiligo patient blood [6]. Higher activity of superoxide dismutase has been demonstrated in both lesional and nonlesional epidermis [7]. Lymphocyte analysis to peripheral blood of patients with vitiligo showed the total levels of T-cells are normal, but the ratio of CD4^+^/CD8^+^ is decreased. The decreased CD4^+^/CD8^+^ ratio of skin-infiltrating T cells and CD8^+^ T cells from vitiligo skin are observed in progressive disease [8]. Significantly higher number of circulation CD8^+^ T cells was shown in progressive generalized vitiligo [9]. Decreased CD4^+^/CD8^+^ ratio was shown in active generalized vitiligo patients, which is involved in the pathogenesis of vitiligo [10]. Increased ROS are thought to be involved in onset of vitiligo, and the infiltration of melanocyte-specific cytotoxic CD8^+^ T cells into the perilesional margin directly result in melanocyte loss [11, 12]. One study [13] reported that oxidative stress leads to chemokine production and causes CD8^+^ T cell skin trafficking and melanocyte destruction in vitiligo. Blockade of oxidative stress can ameliorate melanocyte apoptosis through anti-inflammatory and antiapoptotic processes. CXC chemokine ligand10 (CXCL10) was highly expressed in the skin and serum of patients with vitiligo and is critical to the progression and maintenance of depigmentation in a mouse model of vitiligo. CXCL10-CXCR3 (CXC chemokine receptor 3) axis is critical to both the progression and maintenance of depigmentation in vitiligo mouse models [14, 15].

Antrodia camphorate is a mushroom growing on camphor tree in Taiwan forests. It is a traditional Chinese herbal medicine with several pharmacological effects, such as antioxidant and free radical-scavenging activities [16, 17] and inhibition of inflammatory responses [18, 19]. Antroquinonol is a major active component of Antrodia camphorate and was identified with its anti-inflammatory activity and anticancer potential [20–22]. Antroquinonol displayed anticancer potential for human hepatocellular carcinoma cells by adenosine 5′-monophosphate- (AMP-) activated protein kinase (AMPK) and mammalian target of rapamycin (mTOR) pathways [23] and could protect the kidney from immunologic damage via blocking tumor necrosis factor-*α* (TNF-*α*) and interleukin-1*β*- (IL-1*β*-) mediated inflammatory process [24]. Antroquinonol differentially modulates T cell activity and reduced IL-18 production of murine accelerated severe lupus nephritis [25]. However, it remains to be determined whether antroquinonol is capable of preventing the various depigmentation histopathologic features of C57BL/6 mice treated by hydrogen peroxide (H_2_O_2_). Immunosuppressive effect of antroquinonol on CD8^+^ T cells is still unknown.

We hypothesize that antroquinonol might exert immunosuppressive effect on CD8^+^ T cell proliferation and activation to resist depigmentation induced by H_2_O_2_. To test this, we investigated effects of antroquinonol on depigmentation model induced by H_2_O_2_ that mimics vitiligo *in vivo*.

## 2. Materials and Methods

### 2.1. Study Subjects

This study was approved by the ethics committee of the third people's hospital of Hangzhou. Twenty healthy control's blood samples (Table 1) whose CD^+^ T cells are outrange of reference were collected randomly from physical examination center of the third people's hospital of Hangzhou. Informed consent was obtained, and this study was approved by local ethics committees.

### 2.2. Animals and Treatment

Four-week-old female pathogen-free C57BL/6 mice (weighing 18–20 g) were purchased from Changzhou Cavens Experimental Animal Co. Ltd. (Changzhou, Jiangsu, China) and fed in the laboratory animal research center of Zhejiang Chinese medical university. Mice were housed in groups under specific pathogen-free conditions (22 ± 2°C, RH 50–60%, and a 12 h light/dark cycle). Each mouse was individually weighed and randomly assigned to an experimental group. The mice were housed in polycarbonate cages and fed a standard animal diet with water. All mice were treated in strict accordance with the Zhejiang Chinese Medical University Animal Care and Use committee's guidelines for the care and use of laboratory animals. Before treatment, the back skin of all mice was shaved (area: 2 × 2 cm) and a depilatory cream (Veet, London, UK) was applied to areas. This is aimed to promote hair follicle transferred from telogen stage to anagen stage. Mice were grouped into three: One group of mice was smeared with 1 ml of PBS as control. One group of mice was smeared with 1 ml of 5% H_2_O_2_ in the experimental skin area for 3 minutes at 3 pm. The third group of mice was administered with antroquinonol at 50 mg/kg per day by intragastric administration at 9 am, and H_2_O_2_ was smeared at 3 pm. The mice were treated once per day for continuous 50 days and shaved daily. Three mice were used in one group.

### 2.3. Measurement of Hair Growth, Skin Thickness, and Pigmentation

The distance from the dermal papilla to the epidermis was measured using straight line as hair follicle (HF) length. The width of the surface of the epidermis to the muscle in the photomicrograph was measured as skin thickness. Irregular shape simulated the depilation area, and repigmentation percentage was estimated. All data were normalized to the controls and analyzed statistically.

### 2.4. Antibodies and Reagents

The primary antibodies for immunostaining against CXCL10 (ab8098), CXCR3 (ab71864), tyrosinase (ab54447), and CD8 antibody (ab25478) were purchased from Abcam (Cambridge, USA). ELISA kits for testing interleukin-2 (IL-2) and interferon-*γ* (IFN-*γ*) were obtained from R&D system (Minneapolis, USA). Antibodies for detecting CD69 (MHCD6918) and CD137 (11-1379-42) were purchased from eBioscience (eBioscience, CA, USA). Positive selection using magnetic beads coated with an anti-CD8 monoclonal antibody was purchased from Miltenyi (Bergisch Gladbach, Germany). Antroquinonol was purchased from Golden Biotechnology (Beijing, China).

### 2.5. Preparation of CD8^+^ T Lymphocytes

Peripheral blood mononuclear cells (PBMC) were isolated by density centrifugation using lymphocyte separation media (Mediatech, Herndon, VA) according to the manufacturer's instructions. CD8^+^ T cells were isolated from PBMC by positive selection using magnetic beads coated with an anti-CD8 monoclonal antibody.

### 2.6. CD8^+^ T Cell Proliferation Assay

CD8^+^ T cells were washed in PBS and immediately labeled by incubation with 10 *μ*M CFSE (5-(and-6)-Carboxyfluorescein Diacetate, Succinimidyl Ester) (Invitrogen, Life Technologies Corporation, Saint-Aubin, France) in PBS for 30 minutes at 37°C. After CFSE labeling, CD8^+^ T cells were cultured in 96-well plates coated with anti-CD3 and anti-CD28 for various conditions treated with various dosage of antroquinonol (0, 1.25, 2.5, 5.0, 10, 20, and 40 *μ*M) or different time points (0, 12, 24, 48, and 96 h). After completion of respective incubation time, cells were harvested and washed in PBS. The proliferation of CD8^+^ T cells was evaluated by flow cytometry. Each group was triplicated.

### 2.7. ELISA

The concentrations of IL-2 and IFN-*γ* in collected mice serum and cell culture supernatant were quantified using enzyme-linked immunosorbent assay (ELISA) kits (R&D Systems, Minneapolis, USA) by following the manufacturer's instructions. The absorbance at 405 nm was recorded using a microplate reader. The experiments were repeated for 3 times.

### 2.8. Flow Cytometry

After the different experimental conditions mentioned above, the cells were resuspended in 300 *μ*l of 1x PBS and stained with FITC-labelled CD69 (CH/4) and CD137 ((4-1BB)) for 20 min at 4°C. Then, the cells were fixed in 1% paraformaldehyde for further analysis. After incubation and washing, cells were resuspended in 1x PBS and analyzed by FACSCanto II flow cytometer (BD Biosciences, San Diego, CA, USA). The experiments were repeated for 3 times.

### 2.9. Immunohistochemistry

For immunohistochemistry, skin sections of mice placed on slides (MASCOAT, Matsunami, Osaka, Japan) were deparaffinized with immersion in dimethylbenzene, rehydrated, heated in citrated buffer (0.01 M, pH 6.0) for 5 min at 100°C, and then treated with endogenous peroxidase (3% hydrogen peroxide solution) for 5 min at room temperature. After blocking in 10% goat serum for another 1 h at room temperature, the sections were immunostained with primary antibodies for CXCL10, CXCR3, and tyrosinase diluted in 0.01 M PBS containing 0.3% (*v*/*v*) Triton X-100 and 5% bovine serum albumin overnight at 4°C. The sections were washed with 0.01 M PBS, incubated with biotinylated anti-rabbit IgG before being incubated with the avidin-biotin-peroxidase complex for 30 min at room temperature, and finally visualized using aminoethyl carbazole (AEC) as a peroxidase substrate. Images were captured under an Olympus BX51 microscope installed with ImageJ software.

### 2.10. Immunofluorescence

To detect CD8^+^ T localization, frozen sections of the mice skin were washed with 0.01 M PBS, preincubated with 10% normal goat serum in 0.01 M PBS for 30 min, and then incubated overnight at 4°C with rabbit anti-CD8^+^ T polyclonal antibody (1 : 1000 dilution) in the following solution: 10% normal goat serum in 0.01 M PBS with 0.3% (*v*/*v*) Triton X-100. Sections were washed with 0.01 M PBS, preincubated with 10% normal rabbit serum in 0.01 M PBS for 30 min, and then incubated overnight at 4°C with goat anti-rabbit polyclonal antibody (1 : 5000 dilution) in the following solution: 10% normal rabbit serum in 0.01 M PBS with 0.3% (*v*/*v*) Triton X-100. They were washed with 0.01 M PBS and then incubated for 3 h at room temperature with a mixture of Alexa Fluor 546F(ab′)2 fragment of goat anti-rabbit IgG (H + L) (1 : 1000 dilution) (Molecular Probes). The slips were washed 5 min for 3 times in PBS and mounted using a mounting medium and observed with confocal laser scanning microscope (TCS SP2, Leica, Germany).

### 2.11. Statistical Analysis

SPSS13.0 software (SPSS, Chicago, IL) was employed for statistical analysis. The data are presented as the mean ± SD. One-way analysis of variance (ANOVA) was performed for comparing means across multiple groups. *P* values less than 0.05 were considered statistically significant.

## 3. Results

### 3.1. Effects of Antroquinonol on Proliferation of Human CD8^+^ T Cells

**Figure 1:**
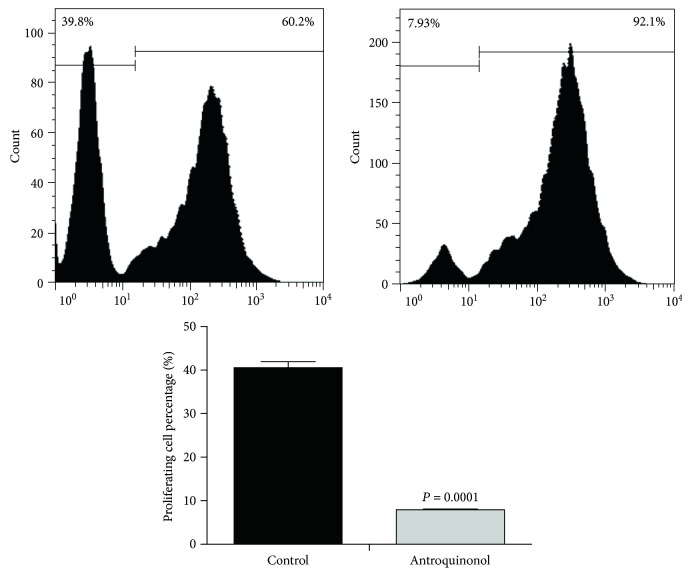
Effects of antroquinonol on proliferation of CD8^+^ T cells. CD8^+^ T cells were cultured with antroquinonol at 20 *μ*M for 48 h. The cellular proliferation was determined by CFSE. The value is shown as mean ± SD. (*n* = 3). *P* < 0.05 is regarded as statistical difference.

To determine the effect of antroquinonol on proliferation of human CD8^+^ T cells, a CFSE assay was performed quantificationally. CD8^+^ T cells were treated with antroquinonol (0–40 *μ*M) for 48 h, and the results indicated that antroquinonol exhibited inhibition in CD8^+^ T cell proliferation. Treatment of antroquinonol at 20 *μ*M showed 35% growth inhibition, and treatment of antroquinonol at 20 and 40 *μ*M indicated similar inhibitory effect on cell proliferation. Compared with control, treatment of antroquinonol at 20 *μ*M for 48 h effectively enhanced the proliferation by 4 times (*P* = 0.0001). Whereas, similar increase at 20 *μ*M for 48 h and 96 h was observed (data not shown). Taken together, the results suggested that treatment of antroquinonol at 20 *μ*M for 48 h was used for following experiments (Figure 1).

### 3.2. Antroquinonol Reduced Production of Cytokines in Human CD8^+^ T Cells

**Figure 2:**
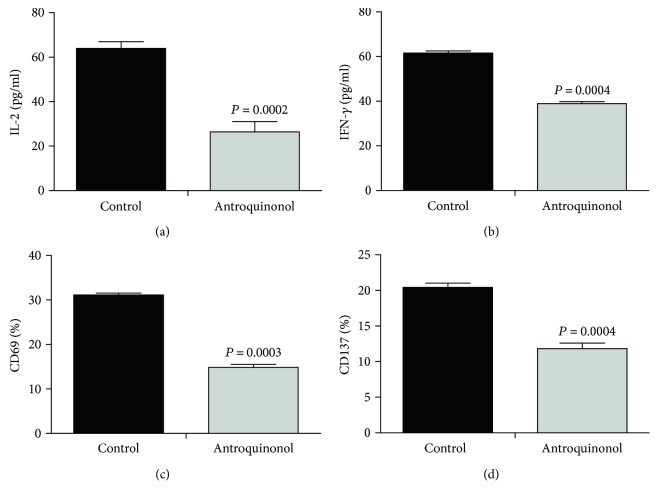
Effects of antroquinonol on cytokine production and T cell activation marker expression of CD8^+^ T cells. CD8^+^ T cells were stimulated with anti-CD3/anti-CD28 in the absence or presence of antroquinonol (20 *μ*M) in a 24-well plate, and the culture supernatants were collected at 48 h for measuring the levels of IL-2 and IFN-*γ* by ELISA, and the expression of CD69 and CD137 by flow cytometry. Levels of IL-2 (a), IFN-*γ* (b), CD69 (c), and CD137 (d) in the antroquinonol-treated CD8^+^ T cells were less than those in the untreated CD8^+^ T cells. The values are presented as mean ± SD. (*n* = 3). *P* < 0.05 means statistical difference.

To investigate the effect of antroquinonol on the production of cytokines associated with CD8^+^ T cells, levels of IL-2 and IFN-*γ* were analyzed by ELISA (Figure 2). The amounts of IL-2 (26.43 ± 4.63 pg/ml) and IFN-*γ* (38.87 ± 0.88 pg/ml) in the antroquinonol-treated CD8^+^ T cells were significantly lower compared with those in the control group IL-2 (63.98 ± 2.98 pg/ml) (*P* = 0.0002, Figure 2(a)) and IFN-*γ* (61.52 ± 0.96 pg/ml) (*P* = 0.0004, Figure 2(b)). Additionally, as activator of CD8^+^ T cells, CD69 and CD137 play an important role in CD8^+^ T cell activation. Therefore, we also examined the levels of CD69 and CD137. The results demonstrated that the concentration of CD69 (14.87 ± 0.67) and CD137 (11.83 ± 0.78) was less in the CD8^+^ T cells treated with antroquinonol than that in the control CD69 (31.16 ± 0.40) (*P* = 0.0003, Figure 2(c)) and CD137 (20.43 ± 0.60) (*P* = 0.0004, Figure 2(d), Supplemental figure 1).

### 3.3. Mice Observation

**Figure 3:**
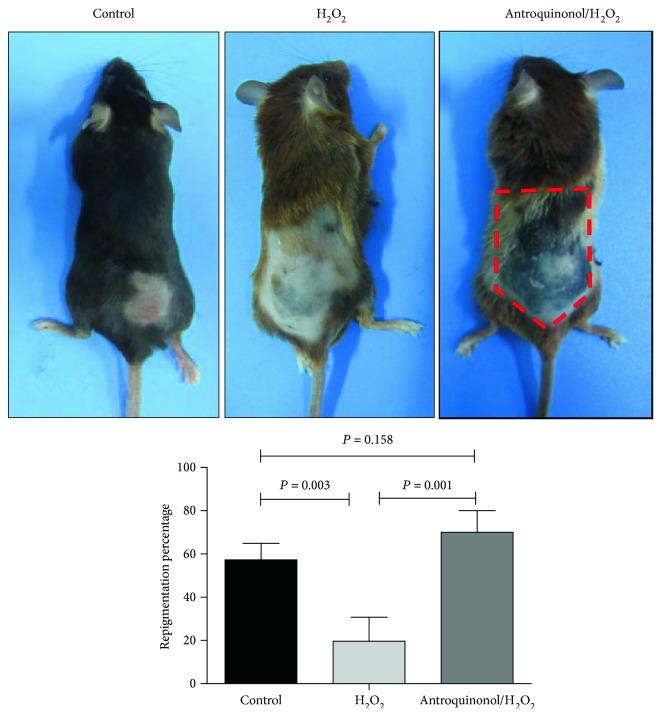
Evaluation of mice treated with H_2_O_2_ and/or antroquinonol. Mice with different treatment for consecutive 50 days were observed. Pigment islands were observed in about 57% of the experimental area, and black hair grew from the pigment islands in the control group. Pigment islands were observed in about 70% of the experimental area, and black hair grew from the pigment islands in the antroquinonol/H_2_O_2_ group. Whereas, a little of pigment islands in the experimental area of the H_2_O_2_ group was shown, and few black hair grew from the pigment islands. The values are presented as mean ± SD. (*n* = 3). *P* < 0.05 means statistical difference.

The pigmentation and hair growth of mice treated with antroquinonol were evaluated. In the antroquinonol/H_2_O_2_ group, pigment islands were observed in about 70% of the experimental area and black hair grew from the pigment islands. In the control group, pigment islands were observed in about 57% of the experimental area and black hair grew from the pigment islands. Whereas, a little of pigment islands in the experimental area of the H_2_O_2_ group were shown and few black hair grew from the pigment islands (Figure 3). This indicated that H_2_O_2_ could induce depigmentation, whereas antroquinonol could inhibit the induction of H_2_O_2_ in depigmentation.

### 3.4. Antroquinonol Resists Inhibition of Hair Growth and Skin Thickness Induced by H_2_O_2_

**Figure 4:**
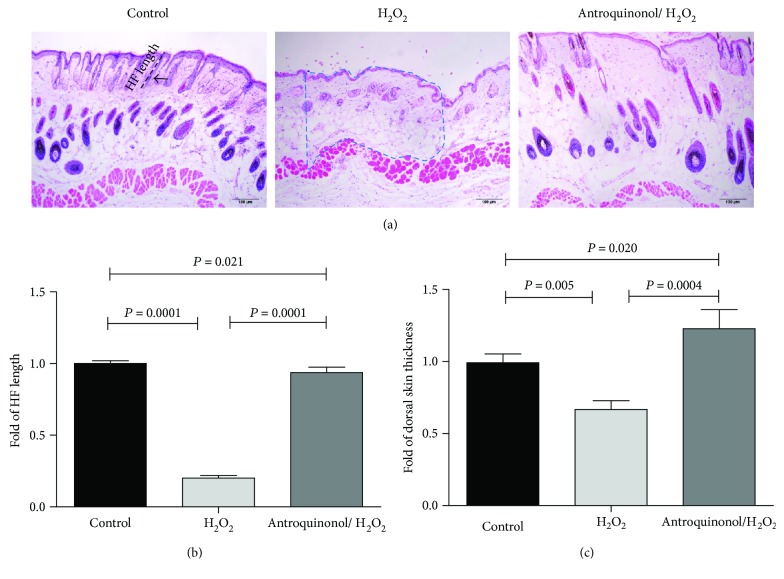
Antroquinonol counteracted inhibition of HF length and skin thickness induced by H_2_O_2_. (a) H&E staining was performed on skin samples harvested after 50 days. HF length and skin thickness were measured. The black dotted line represented the HF. The area within the blue dotted line represents the skin thickness. The black arrows indicated the hair shaft. Scale bar = 100 *μ*m. (b) HF length is presented as the mean length of all photomicrographs ± SD. (c) Dorsal skin thickness is presented as the mean thickness of all photomicrographs ± SD (*n* = 3). *P* < 0.05 means statistical difference.

To investigate the role of antroquinonol on the growth of hair and skin, we performed H&E staining to visualize hair follicle length and skin thickness (Figure 4(a)). On the 50th day after depilation, the hair follicle length of the mice in the control group (*P* = 0.0001) and the antroquinonol/H_2_O_2_ group (*P* = 0.0001) was significantly larger compared to the mice in the H_2_O_2_ group (Figure 4(b)). Similarly, skin thickness in the control group (*P* = 0.005) and the antroquinonol/H_2_O_2_ group (*P* = 0.0004) was significantly higher than that in the H_2_O_2_ group (Figure 4(c)). Collectively, antroquinonol could resist inhibition of hair growth and skin thickness induced by H_2_O_2_.

### 3.5. Antroquinonol Induced Expression of Tyrosinase

**Figure 5:**
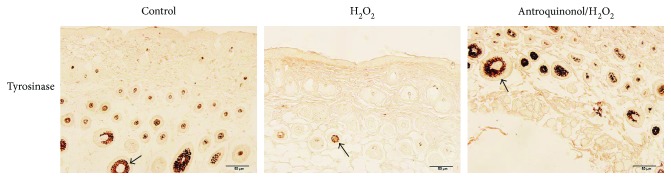
Antroquinonol resisted decrease of tyrosinase induced by H_2_O_2_. Skin sections were examined with immunohistochemistry staining with anti-tyrosinase antibody. Contrast to the control group, lower expression of tyrosinase was observed in the H_2_O_2_ group, and a little higher expression of tyrosinase was shown in the antroquinonol/H_2_O_2_ group. The black arrows indicated the hair follicle. Scale bar = 50 *μ*m.

Tyrosinase is the key enzyme of melanogenesis. We detected its expression in the skin with immunohistochemistry (Figure 5). The results showed that the expression of tyrosinase was obviously reduced in the H_2_O_2_ group. In the control group, amounts of tyrosinase are mostly expressed in the hair follicle. Similarly, much tyrosinase was detected in the antroquinonol/H_2_O_2_ group. This indicates that H_2_O_2_ could inhibit the expression of tyrosinase, whereas antroquinonol could resist the inhibition of H_2_O_2_ to the induction of tyrosinase.

### 3.6. Antroquinonol Could Inhibit Infiltration of Mouse CD8^+^ T Cells

**Figure 6:**
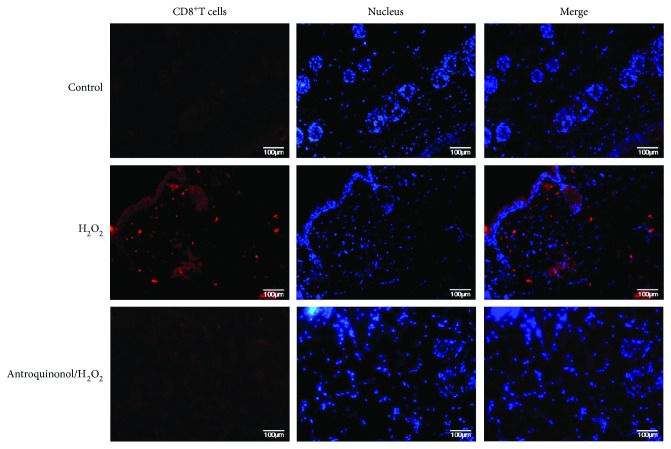
Antroquinonol attenuated infiltration of CD8^+^ T cells induced by H_2_O_2_. Skin sections were examined with immunofluorescence staining for CD8^+^ T cells. The cell surface markers, CD8, was identified in the left column of the figure. Middle column was detected for nucleus counterstaining with DAPI. Right column was merged image. The control group showed only few CD8^+^ T cell infiltration. Similarly, a few of CD8^+^ T cells were observed in the antroquinonol/H_2_O_2_ group. Whereas numerous CD8^+^ T cells infiltrated in the skin of the H_2_O_2_ group. Scale bar = 100 *μ*m.

In order to investigate whether antroquinonol exert immunosuppressive effect on CD8^+^ T cells, immunofluorescence assay was performed to detect the infiltration of CD8^+^ T cells. As shown in Figure 6, amount of CD8^+^ T cells were observed in the experimental area in the H_2_O_2_ group. A few of CD8^+^ T cells were shown in the skin in the antroquinonol/H_2_O_2_ group. Few CD8^+^ T cells were detected in the control group. This indicated that H_2_O_2_ could enhance the infiltration of CD8^+^ T cells, whereas antroquinonol could inhibit the infiltration of CD8^+^ T cells induced by H_2_O_2_.

### 3.7. Antroquinonol Reduced Production of IL-2 and IFN-*γ*

**Figure 7:**
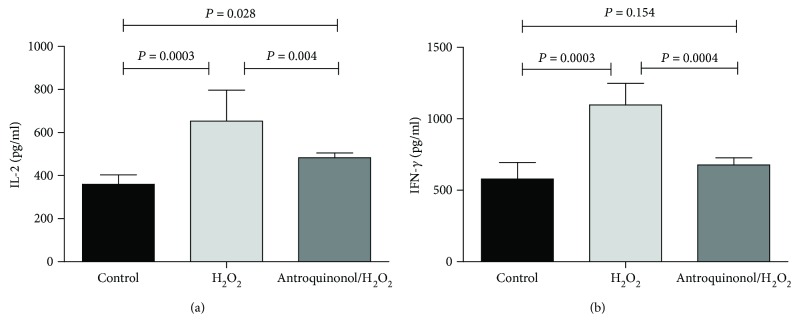
Effects of antroquinonol on cytokine production in mice. Separated sera were collected from mice blood and measured IL-2 and IFN-*γ* by standard ELISA protocols. (a) Level of IL-2. (b) Level of IFN-*γ*. Contrast to the H_2_O_2_ treated mice, decreased levels of IL-2 and IFN-*γ* were shown in the antroquinonol-treated mice and the untreated mice. Levels of IL-2 and IFN-*γ* were higher in the antroquinonol-treated mice than those in the untreated mice. The values are presented as mean ± SD (*n* = 3). *P* < 0.05 means statistical difference.

Production of cytokine IL-2 and IFN-*γ* was determined with ELISA (Figure 7). Among the three groups, the lowest level of IL-2 (359.50 ± 43.85 pg/ml) and IFN-*γ* (578.46 ± 115.69 pg/ml) was detected in the control group, and the highest level of IL-2 (653.00 ± 144.07 pg/ml) and IFN-*γ* (1096.93 ± 151.55 pg/ml) was detected in the H_2_O_2_ group. Significant difference of IL-2 (*P* = 0.0003) and IFN-*γ* (*P* = 0.0002) between the control group and the H_2_O_2_ group was observed. Significance between the level of IL-2 (482.67 ± 22.62 pg/ml) (*P* = 0.028) and IFN-*γ* (677.20 ± 49.84 pg/ml) (*P* = 0.154) in the antroquinonol/H_2_O_2_ group and that in the control group was observed, but significantly lower level of IL-2 (*P* = 0.004) and IFN-*γ* (*P* = 0.0004) than that in the H_2_O_2_ group. It indicated that H_2_O_2_ could promote the production of IL-2 and IFN-*γ*, but antroquinonol could ameliorate the effect of H_2_O_2_.

### 3.8. Antroquinonol Could Reduce Expression of Chemokine CXCL10 and Its Receptor CXCR3

**Figure 8:**
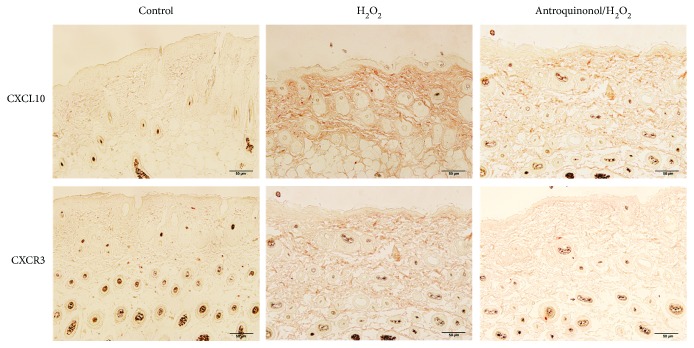
Antroquinonol decreased the expression of CXCL10 and CXCR3 induced by H_2_O_2_. Skin sections were examined with immunohistochemistry staining with anti-CXCL10 and anti-CXCR3 antibodies. Contrast to the control group, obvious high expression of CXCL10 and CXCR3 was detected in the H_2_O_2_ group, and a little higher expression of CXCL10 and CXCR3 was observed in the antroquinonol/H_2_O_2_ group. Scale bar = 50 *μ*m.

Immunohistochemistry was performed to investigate the expression of CXCL10 and CXCR3. As demonstrated in Figure 8, high expression of CXCL10 and CXCR3 was observed in the H_2_O_2_ group. Contrast to the H_2_O_2_ group, obviously reduced expression of CXCL10 and CXCR3 was observed in the antroquinonol/H_2_O_2_ group. The expression of CXCL10 and CXCR3 was lower in the mice of the control group. This indicated that H_2_O_2_ could promote the expression of CXCL10 and CXCR3, whereas antroquinonol could inhibit the increase of CXCL10 and CXCR3 induced by H_2_O_2_.

## 4. Discussion

Vitiligo is a common dermatological disorder of the epidermis characterized by the acquired loss of melanocytes and melanin. The interplay between oxidative stress and the immune system plays significant roles in the pathogenesis of vitiligo. Increased evidence supported that oxidative stress plays a critical role in the autoimmune initiation in vitiligo [2, 26]. Higher level of H_2_O_2_ was demonstrated in vitiligo epidermis than that in healthy controls [4]. Here, we induce depigmentation with H_2_O_2_ in mouse to simulate vitiligo. 5% H_2_O_2_ was applied to smear topically in the skin of mice for inducing depigmentation [27]. After 50 days, mice in the H_2_O_2_ group showed white skin in the experimental area and yellow hair grew from the experimental area. This indicated that H_2_O_2_ could induce depigmentation. In further, H&E staining was applied to investigate the hair follicle length and skin thickness in the experimental area. In the H_2_O_2_ group, hair follicle length and skin thickness were significantly lower than those in the control group. In mice, melanocytes grow in hair follicles which provide lieu to melanocyte survival and subsequent melanogenesis. Inhibition of hair follicle growth suppresses biological activity of melanocyte. Tyrosinase has a key role in pigmentation process, and which could be impacted by a range of materials on its activity. Tyrosinase activity in vitiligo patients' lesional skins was lower than that in vitiligo patients' nonlesional skins [28]. In this study, tyrosinase expression is dramatically decreased in the mice treated with H_2_O_2_, which is similar to that in vitiligo patients' lesional skins. Together, it indicates that mice treated with H_2_O_2_ could simulate vitiligo patients. Therefore, we used this model to detect antroquinonol effect on the vitiligo.

Several biological activities of natural food-derived components were reported for their promising anti-inflammatory, antioxidant, and antiapoptotic modulatory potential [29–31]. Flavonoids present in fruits, vegetables, and herbs exert a positive health effect in neurodegenerative disorders and cancer, owing to their free radical-scavenging activities [32]. Antioxidants, oral vitamins, and supplements have also gained increased interest in the treatment of vitiligo for their antioxidant properties. *Ginkgo biloba*, resveratrol, and zinc have all been studied either as monotherapies or in combination with other treatments with varying efficacy in improving vitiligo repigmentation [33–36]. Our previous study also showed that quercetin (3,5,7,3′,4′, pentahydroxyflavone) could attenuate the effects of H_2_O_2_ on the tyrosinase export from the endoplasmic reticulum in melanocytes [37].

Antrodia camphorata, a parasitic fungus on rotting trees of Cinnamomum kanehirai Hay in Taiwan [20], which is used as a folk medicine and has been shown to have several pharmacologic effects, including antioxidant and free radical-scavenging activities [16], inhibition of the inflammatory response [19], and antitumor cytotoxicity activity [38]. Antroquinonol, a major active component of Antrodia camphorata, has been shown to inhibit T cell activation/proliferation and production of ROS and suppress NF-*κ*B activation and NF-*κ*B-dependent inflammation and activation of Nrf2 [25, 39]. In this study, we provide the first demonstration that antroquinonol can inhibit the CD8^+^ T cell infiltration and reduced tyrosinase induced by H_2_O_2_.

Firstly, we investigate the function of antroquinonol on human CD8^+^ T cells *in vitro*. About 20 *μ*M of antroquinonol was incubated in human CD8^+^ T cells for 48 h. The results showed antroquinonol could inhibit CD8^+^ T cell proliferation and activation of CD8^+^ T cells by suppressing production of CD69, CD137, IL-2, and IFN-*γ*. And then in *vivo* investigation was performed. The results indicated that antroquinonol could suppress the proliferation and production of cytokines of CD8^+^ T cells. Moreover, effect of antroquinonol on CD8^+^ T cells in mice treated with H_2_O_2_ was detected. In the antroquinonol/H_2_O_2_ group, pigment islands were observed in 80% of the experimental area and black hair grew from the pigment islands. In the control group, pigment islands were observed in 50% of the experimental area and black hair grew from the pigment islands. Whereas, in the H_2_O_2_ group, a little of pigment islands in the experimental area was shown and few black hair grew from the pigment islands. This indicated that H_2_O_2_ could induce depigmentation, whereas antroquinonol could inhibit the induction of H_2_O_2_ in depigmentation. In further, H&E staining was applied to investigate the hair follicle length and skin thickness in the experimental area. In the H_2_O_2_ group, hair follicle length and skin thickness were significantly lower than those in the antroquinonol/H_2_O_2_ group and the control group. There was no significant difference of hair follicle length between the antroquinonol/H_2_O_2_ group and the control group. Skin thickness in the antroquinonol/H_2_O_2_ group was higher than that in the control group. Expression of tyrosinase was examined in all groups. In the H_2_O_2_ group, a little of tyrosinase was observed in the hair follicle. Contrast to the H_2_O_2_ group, increased expression of tyrosinase was detected in the control group and the antroquinonol/H_2_O_2_ group. These results showed that antroquinonol could promote hair follicle growth, expression of tyrosinase, and repigmentation. It indicates that antroquinonol could be a potential candidate for interference in depigmentation.

In vitiligo, CD8^+^ T cells are involved in autoimmune responses, resulting in depigmentation of the skin [40]. Cytokines released by lymphocytes, including IL-1, IFN-*γ* or TNF-*α*, can initiate apoptosis of both melanocytes and keratinocytes [41, 42]. IFN-*γ*, as one important cytokine associated with the Th1 immune response, induced protein CXCL10 to express in various cell types, such as lymphocytes, fibroblasts, neutrophils, and other epithelial cells. Some studies have proposed that IFN-*γ*–induced CXCL10–CXCR3 chemokine pathway plays a vital role in CD8^+^ T cell skin infiltration [14, 15, 41, 43]. CXCL10 binds to its specific receptor CXCR3 to recruit and activate T cells for regulating immune responses. Increased expression of CXCL10 and CXCR3 was shown in various autoimmune diseases, and they play fundamental parts in leukocyte homing into the inflamed tissues to accelerate the process of tissue damage [44, 45]. Highly induced CXCL10 and CXCR3 were found in vitiligo patients [14]. Here, cytokines IL-2 and IFN-*γ* were examined with ELISA. H_2_O_2_ significantly enhanced the level of IL-2 and IFN-*γ* in mice, and antroquinonol could inhibit the production of IL-2 and IFN-*γ*. In further, we investigated the IFN-*γ*-induced expression of CXCL10 and CXCR3. In consistent, highly increased expression of CXCL10 and CXCR3 was found in the mice treated with H_2_O_2_. A little increase expression of CXCL10 and CXCR3 was detected in the mice treated with antroquinonol/H_2_O_2_.

## 5. Conclusions

According to our findings in this study, it is suggested that antroquinonol has a potential therapeutic effect on depigmentation. Antroquinonol significantly attenuated histopathologic changes in the mice skins and inhibited the infiltration of CD8^+^ T cells and expression of chemokines CXCL10 and CXCR3. In addition, antroquinonol could decrease the production of cytokines IL-2 and IFN-*γ* obviously and promote tyrosinase expression. These results suggest that antroquinonol might be a treatment of choice for preventing depigmentation.

## Figures and Tables

**Table 1:** Information of the study subjects.

Sex of subjects	Number	Age	CD8^+^ T cells	Reference range of CD8^+^ T cells
Female	10	36.40 ± 6.28	1564.60 ± 68.01	190–1440
Male	10	37.50 ± 7.15	1535.00 ± 64.46	
Total	20	36.95 ± 6.57	1549.80 ± 66.26	

## Supplementary Materials

Supplementary Materials Effects of antroquinonol on expression of CD8^+^ T cell activation markers. CD8^+^ T cells were stimulated with anti-CD3/anti-CD28 in the absence or presence of antroquinonol (20 μM) in a 24 well plate, and cells were collected at 48 h for measuring the expression of CD69 (a) and CD137 (b) by flow cytometry.
