# Corrigendum to “Antroquinonol Exerts Immunosuppressive Effect on CD8^+^ T Cell Proliferation and Activation to Resist Depigmentation Induced by H_2_O_2_”

**DOI:** 10.1155/2020/1402346

**Published:** 2020-10-19

**Authors:** Cuiping Guan, Qingtian Li, Xiuzu Song, Wen Xu, Liuyu Li, Aie Xu

**Affiliations:** ^1^Department of Dermatology, The Third People's Hospital of Hangzhou, Hangzhou 310009, China; ^2^Department of Medicine, Baylor College of Medicine, Houston, TX 77030, USA

In the article titled “Antroquinonol Exerts Immunosuppressive Effect on CD8^+^ T Cell Proliferation and Activation to Resist Depigmentation Induced by H_2_O_2_” [1], there was an error in Figure 8. The figure should show a little higher expression of CXCL10, and CXCR3 was observed in the antroquinonol/H_2_O_2_ group. The corrected figure is shown below and is listed as Figure 1:

## Figures and Tables

**Figure 1:**
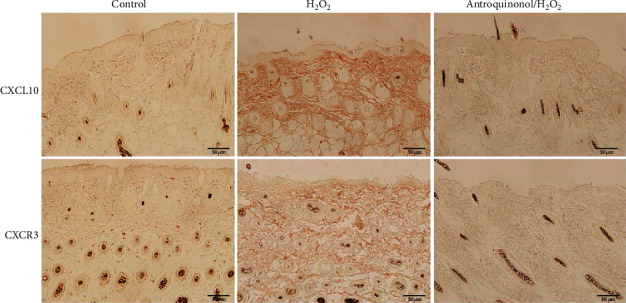
Antroquinonol decreased the expression of CXCL10 and CXCR3 induced by H_2_O_2_. Skin sections were examined with immunohistochemistry staining with anti-CXCL10 and anti-CXCR3 antibodies. Contrast to the control group, obvious high expression of CXCL10 and CXCR3 was detected in the H_2_O_2_ group, and a little higher expression of CXCL10 and CXCR3 was observed in the antroquinonol/H_2_O_2_ group. Scale bar = 50 *μ*m.
