# Supplementary material for: Antroquinonol Exerts Immunosuppressive Effect on CD8+ T Cell Proliferation and Activation to Resist Depigmentation Induced by H2O2
Source: Oxid Med Cell Longev. 2017 Dec 31;2017:9303054. doi: 10.1155/2017/9303054 (PMC5804328; doi:10.1155/2017/9303054)
Supplement: Supplementary Materials — Effects of antroquinonol on expression of CD8+ T cell activation markers. CD8+ T cells were stimulated with anti-CD3/anti-CD28 in the absence or presence of antroquinonol (20 μM) in a 24 well plate, and cells were collected at 48 h for measuring the expression of CD69 (a) and CD137 (b) by flow cytometry. [file 9303054.f1.docx]

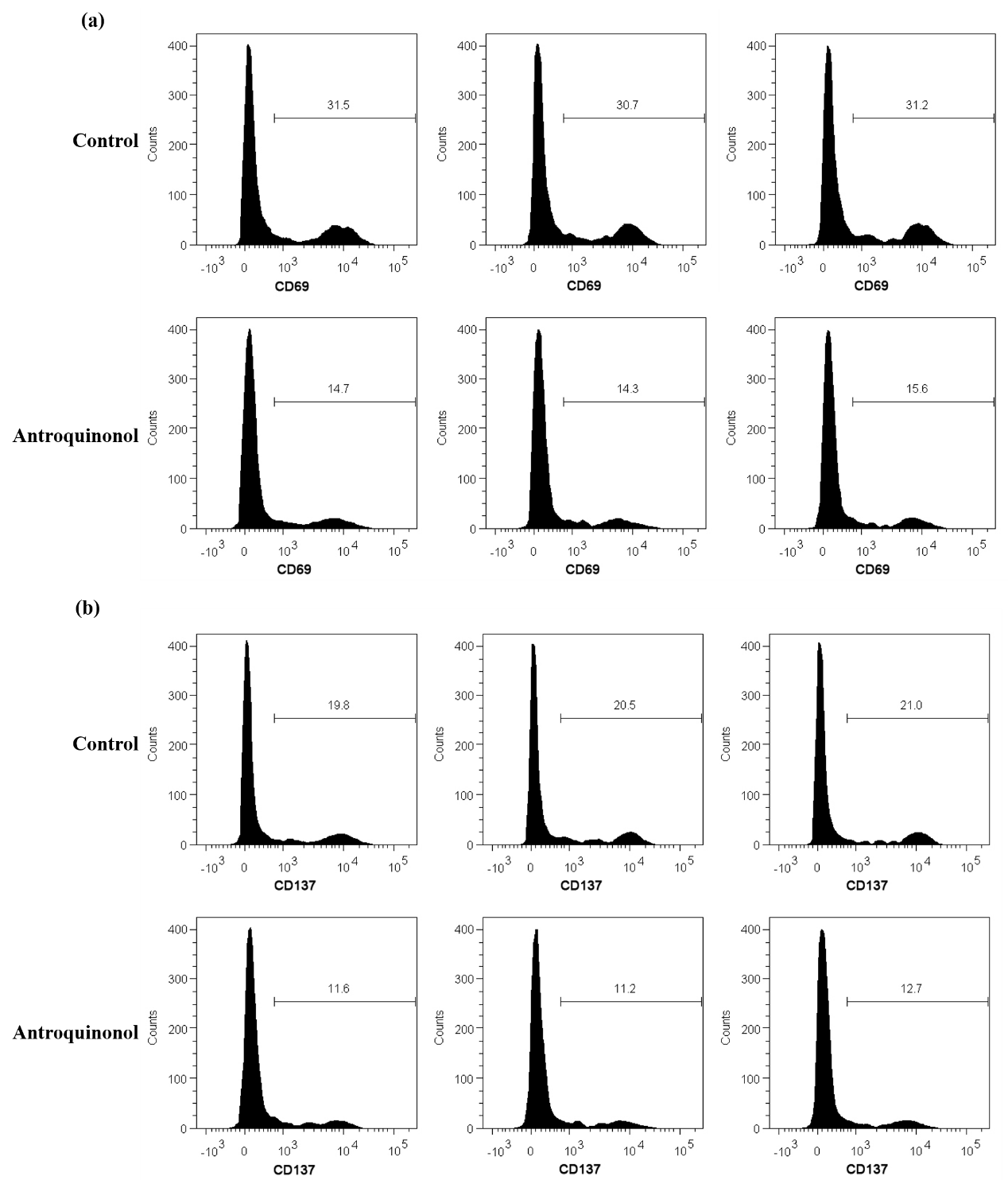


Supplemental figure 1. Effects of antroquinonol on expression of CD8^+^ T cell activation markers. CD8^+^ T cells were stimulated with anti-CD3/anti-CD28 in the absence or presence of antroquinonol (20 μM) in a 24 well plate, and cells were collected at 48 h for measuring the expression of CD69 (a) and CD137 (b) by flow cytometry.
